# Coffee Extends Yeast Chronological Lifespan through Antioxidant Properties

**DOI:** 10.3390/ijms21249510

**Published:** 2020-12-14

**Authors:** Jadwiga Czachor, Michał Miłek, Sabina Galiniak, Karolina Stępień, Małgorzata Dżugan, Mateusz Mołoń

**Affiliations:** 1 Department of Biochemistry and Cell Biology, Institute of Biology and Biotechnology, University of Rzeszow, Zelwerowicza 4, 35-601 Rzeszow, Poland; jadwigac96@gmail.com (J.C.); karolina.stepien89@interia.pl (K.S.); 2 Department of Chemistry and Food Toxicology, Institute of Food Technology and Nutrition, University of Rzeszów, Ćwiklińskiej 1a, 35-601 Rzeszów, Poland; mmilek@ur.edu.pl (M.M.); mdzugan@ur.edu.pl (M.D.); 3 Institute of Medical Sciences, Medical College, University of Rzeszów, Warzywna 1a, 35-959 Rzeszów, Poland; sgaliniak@ur.edu.pl

**Keywords:** aging, antioxidants, budding yeast, coffee, caffeine, longevity, polyphenols

## Abstract

Aging is a multifactorial process accompanied by loss of cell function. Science has been looking for factors responsible for aging for many years. However, despite identifying a number of possible causes, the definite reason for aging has been elusive so far. One of the factors contributing to aging is oxygen free radicals. In this context, beneficial effects of coffee on various organisms, including humans, were investigated, although the results are far from unequivocal. In our research, we used the budding yeast—something of a workhorse in many studies, including the studies of aging. So far, the impact of coffee on the aging of cells in the budding yeast experimental setup has little known about it. Here, we provide strong evidence that coffee compounds, particularly flavonoids, are responsible for scavenging free radicals and longevity in yeast lacking Sod1, Sod2 and Rad52 proteins. In this paper, we compared Arabica and Robusta coffee types. We present an analysis of the concentration of caffeine and flavonoids measured by the High-Performance Liquid Chromatography method. We show that Robusta has a much greater antioxidant capacity than Arabica. We also conclude that coffee infusions significantly extend the chronological lifespan of the *Saccharomyces cerevisiae* yeast cells by protecting cells against reactive oxygen species, double DNA-strand break and decrease in metabolic activity.

## 1. Introduction

Coffee is regarded as one of the world’s most popular beverages with around 2.25 billion cups consumed every day. It is the food product and stimulant preferred by most consumers. A properly prepared coffee drink has a perfect aroma while stimulating, refreshing and enhancing cognitive functions [1]. Coffee is obtained from the tropical bush of the *Coffea* genus. After oil, coffee is the second most frequently traded commodity on international exchanges. The most popular coffee varieties are *Coffea arabica* Linnaeus (Arabica) and *Coffea cenaphora* Pierre (Robusta), which represent respectively 70% and 30% of the global production. As far as quality is concerned, Arabica is appreciated mainly for its better taste and high acidity compared to bitter-tasting while Robusta is famous for its intense dark aroma [2]. Although caffeine is the main chemical compound associated with coffee, the composition of coffee is more complex. Beside caffeine, coffee beans contain polyphenols, diterpenes, lipids, saccharose and proteins. During the bean roasting process, these various aroma precursors are transformed by Maillard reactions. Chlorogenic acids and caffeine are usually responsible for coffee’s bitter taste, while free carbohydrates such as saccharose interact with amino acids creating flavor compounds. Saccharose also lends sweetness to the beverage’s final taste [3]. Thus, the chemical composition of coffee changes in terms of quality and quantity during coffee beans processing. In addition, the coffee roasting process leads to formation of organic acids such as acetic, lactic and formic acids. Coffee also contains vitamins B2 and B3 and minerals such as magnesium, sodium and potassium [4,5].

In the 1960s, coffee beans began to be referred to as a “specialty”, although the term originally applied to coffee blends created from high quality coffee tree beans of the Arabica species. Later, the term was defined in detail by the Specialty Coffee Association of America (SCAA), an organization assessing the quality of coffee. According to the latest guidelines, specialty coffee must score at least 80 of the total 100 scores on the scale of taste and aroma quality [6].

Caffeine (C_8_H_10_N_4_O_2_) is the main active compound [7]. This natural purine alkaloid was found in fruits and leaves of over 60 plants, including coffee, tea and cocoa. In plants, caffeine has a protective function acting as a pesticide: it paralyzes and kills the insect feeding on the plant [8]. The caffeine content of coffee may vary depending not only on the method of brewing but also on the product brand or type of coffee. Caffeine is quickly absorbed in the digestive tract with 100% bioavailability [9]. When consumed, caffeine becomes pharmacologically active within 6–8 min, achieving its maximum concentration level after 30 min. After drinking coffee, caffeine is absorbed by various body tissues. Half-life of this compound is ca. 3–5 h; however, this may be different for different people (pregnant women) or groups (tobacco smokers). Caffeine biotransformation takes place in the liver; around 2% of the caffeine is removed in an unchanged form from the body with urine. The substance is metabolized in the liver by cytochrome P450 enzyme during a metabolic cycle in which three main metabolites are produced: paraxanthine, theophylline and theobromine [10,11,12]. Moreover, the addictive effect of excessive and regular intake was reported [13]. Despite that the acceptable daily intake of caffeine is not set, the daily dose for adult as 200 mg, which corresponds to 3 cups of 100 mL manual dripped coffee, is recommended [14,15].

Coffee owes its health-enhancing qualities to polyphenols due to their antioxidant action. From the chemical point of view, polyphenols are phenol derivatives containing at least two hydroxyl groups attached to the aromatic ring. Depending on the number of aromatic rings and the way in which they are connected polyphenols can be classified as phenolic acids, flavonoids, stilbenes and lignans. Coffee’s antioxidant properties are contributed by its large number of hydroxyl groups, which protect the organism from adverse impact of oxygen free radicals. Some polyphenols also show anti-inflammatory, anticoagulant, antifungal and antiviral properties and blood vessel sealing action. Polyphenolic compounds are also responsible by coffee’s characteristic flavor and aroma. Roasted coffee beans contain ca. 8% of polyphenolic compounds such as caffeic acid, chlorogenic acid and ferulic acid [16].

There are a number of studies showing that consumption of coffee is linked to a lower risk of various types of cancer, type 2 diabetes, ischemic stroke, Alzheimer’s disease and Parkinson’s disease. As shown by the latest research, coffee not only improves concentration and motor skills; it also has antiaging properties [17,18]. It must be stressed, however, that beside many beneficial properties mentioned in this article coffee may also have harmful effects during excessive intake. Excessive consumption of caffeine may lead to an increased blood level by narrowing blood vessels, and prolonged consumption of large caffeine doses, especially when combined with energy drinks, may lead to problems with arteries. Furthermore, as shown by studies on patients with asthma, caffeine overuse may impair bronchodilation up to 4 h [19]. In children, caffeine may cause rapid mood changes and disturb the organism’s calcium metabolism [20]. There are also reports that high consumption of caffeine may affect the population’s fertility. Studies on rats showed that rats fed on caffeine had lower implantation and live birth rates compared to the control group [21]. It was demonstrated that caffeine is easily passed to breast milk; hence nursing mothers are advised to control their caffeine intake [22].

Although the contemporary medicine and biogerontological studies provide some answers to the question of how aging may be partially delayed or inhibited, the problem still remains unresolved and continues to spark general interest. Aging is a multifactorial process leading to loss of function of cells, tissues, organs and ultimately the function of the whole body. Due to the process complexity, its background has not yet been determined, and no specific factor determining aging of organisms or cells has been identified despite substantial efforts. The term “aging” refers to a series of undesirable changes that affect efficiency of life processes and lead to visible structural changes in the organism resulting with its death [23]. Studies of aging are based on model organisms, including unicellular eukaryotes such as the *Saccharomyces cerevisiae* yeast. The budding yeast has been regarded as an acceptable model organism for many years. It has been widely used in studies of replicative and chronological aging, the former describing the number of daughter cells produced by the mother cell during its lifetime [24]. While replicative aging refers to aging of mitotically active cells, the chronological model describes the time of life of yeast cells in the post-mitotic phase; this model is applied to studies of postmitotic cell aging in higher eukaryotes, including humans [25].

Impact of coffee on the aging of cells in the budding yeast experimental setup has little known so far. Therefore, for the purpose of this study we used strains devoid of antioxidative protection (*SOD1* and *SOD2* gene deletion) or DNA damage repair ability (*RAD52*) in order to measure the antioxidative effect of coffee on cells during chronological aging.

## 2. Results

### 2.1. Analysis of Caffeine and Polyphenols in Specialty Coffee Infusions

**Figure 1:**
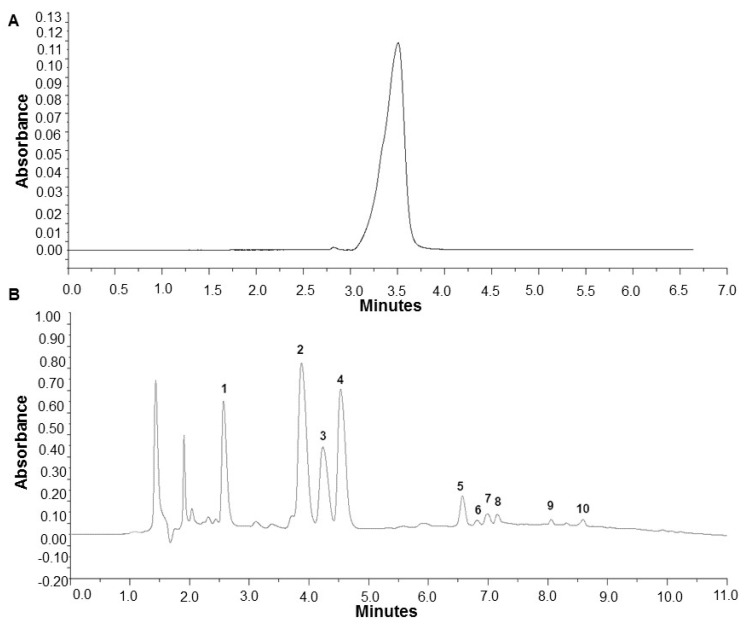
High-Performance Liquid Chromatography with Diode-Array Detection (HPLC-DAD) chromatograms for Arabica coffee infusion. (**A**)—caffeine, (**B**)—polyphenols: 1—neochlorogenic acid, 2—cryptochlorogenic acid, 3—chlorogenic acid, 4—caffeine, 5—ferulic acid, 6–10—dicaffeoylquinic or feruloylquinic acids isomers.

The beneficial properties of coffee result from caffeine and other antioxidants, mainly polyphenols content. Thus in the first step of our study, we determined the caffeine content and polyphenolic profile of the coffee infusions samples, which were next used in the experiment with the yeast model system. For comparison two samples of Arabica and two sample of Robusta specialty grade coffee were used. All quantitative analyses were performed using the (High-Performance Liquid Chromatography) HPLC method; exemplary chromatograms are presented in Figure 1.

It was found that Robusta coffee infusions contained on average significantly more caffeine compared to Arabica infusions, 2.55 mg/mL and 1.63 mg/mL, respectively. Analysis of polyphenol content showed that Robusta has more polyphenols compared to Arabica. Cryptochlorogenic acid made up the largest fraction of polyphenols, followed by chlorogenic acid and neochlorogenic acid (Table 1).

In Arabica coffee cryptochlorogenic and chlorogenic acids were present in almost equal concentrations, neocholorogenic acid content was lower. In the case of Robusta, cryptochlorogenic acid was determined predominantly, the other two isomers were present in lower, comparable concentrations. The content of ferulic acid was also significantly higher in the latter variant. In all samples numerous isomers of dicaffeoylquinic or feruloylquinic acid were detected. An exact identification of these compounds would require the HPLC method with mass detection. The results indicating higher polyphenols content in the Robusta variety were confirmed by the total phenolic content (TPC) analysis. The polyphenol content determined according to this method is 3.11 mg GAE/mL for the Robusta coffee, while it is significantly lower for Arabica amounting to 2.08 mg GAE/mL (Table 2).

### 2.2. Antioxidant Properties of Coffee Infusions

Antioxidant activity is the main parameter by which bioactive compound content is measured in the examined coffee samples. The antioxidant power of Robusta infusion samples determined with the ABTS* radical method and the FRAP method yielded higher antioxidant effects compared to Arabica (Table 1, *p* < 0.05). Similarly, a stronger radical scavenging activity (2,2-diphenyl-1-picrylhydrazyl (DPPH) test) was found in the Robusta infusion. We also showed that caffeine at 1 and 10 mg/mL concentrations displayed very weak (DPPH) or no antioxidant activity (ABTS and FRAP; Table 2).

### 2.3. Coffee Infusion Reduces Yeast Cell Growth

To investigate the effects of coffee on aging, a budding yeast model system was used. Yeast is an acceptable model for studying many physiological processes, including aging and cell death. In these studies, we used the *SOD1*, *SOD2* and *RAD52* mutants in the background of the BY4741 haploid laboratory strain. Mutants lacking superoxide dismutase, cytosolic *SOD1* or mitochondrial *SOD2* are involved in detoxification of the superoxide anion radical in the cell, while the Rad52 protein is involved in repairing DNA double-strand breaks and maintaining genome stability. 

**Figure 2:**
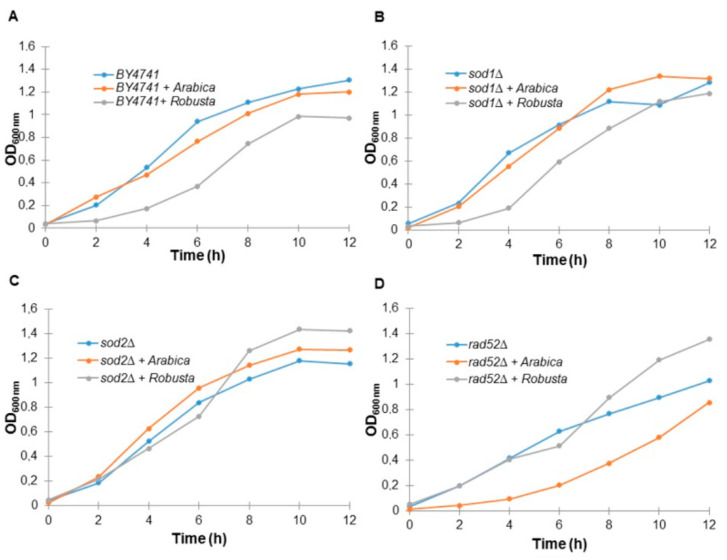
Comparison of growth kinetics of the haploid wild-type yeast strain BY4741 (**A**) and isogenic mutant strains *sod1Δ* (**B**), *sod2Δ* (**C**) and *rad52Δ* (**D**) treated with coffee infusion. The optical density (OD_600_) of the cultures was measured at different time points for up to 12 h. The presented data are replicates from two independent cultures ± SD where these are smaller or the same size as the symbol dimensions. The data for each condition are representative of at least two independent experiments performed on different days.

First, we showed that *S. cerevisiae* yeast cells treated with coffee infusions had a reduced growth rate. As shown in Figure 2, almost all of the analyzed strains had a reduced growth rate, especially in the case of the wild-type strain and the *sod1Δ* and *rad52Δ* mutants. Interestingly, Arabica coffee infusions inhibited growth of the wild-strain and *sod1Δ* strains much more strongly, while in the case of *rad52Δ* Robusta had a much greater impact on the reduction of the growth rate. In turn, in the case of the *sod2Δ* mutant, we did not see a spectacular effect of coffee on growth.

### 2.4. Impact of Coffee on Saccharomyces Cerevisiae Yeast Aging

The most recent reports provide a vast amount of data on the health and antiaging effects of coffee on model organisms and humans. Therefore, as a next step we analyzed the effect of coffee infusions on yeast chronological lifespan. Chronological lifespan of yeast was measured as the yeast population’s survival during the stationary phase or post-diauxic phase [25].

**Figure 3:**
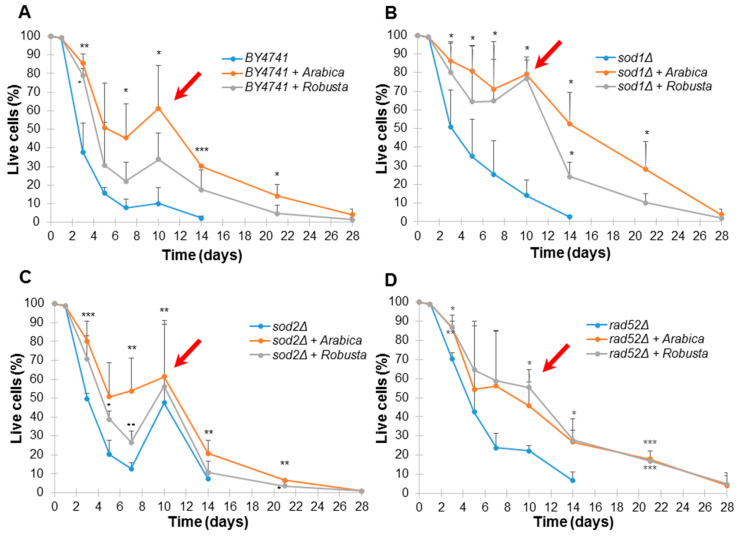
Chronological lifespan of the haploid wild-type yeast strain BY4741 (**A**) and isogenic mutant strains *sod1Δ* (**B**), *sod2Δ* (**C**) and *rad52Δ* (**D**) treated with Arabica and Robusta infusions coffee. Survival was determined by propidium iodide staining. Statistical significance was assessed using ANOVA and the Dunnett’s post hoc test (* *p* < 0.05; ** *p* < 0.01, *** *p* < 0.001) compared to the control (untreated). Bars indicate SD. Red arrows show adaptative regrowth.

As shown in Figure 3, coffee infusion significantly delays aging of all yeast strains used in this study. Interestingly, Arabica infusions inhibited aging more strongly in the case of the wild-type strain and the *sod1Δ and sod2Δ* mutants, while Robusta had a stronger antiaging effect on the *rad52Δ* strain.

The Arabica coffee had a statistically significant effect on the chronological aging of the wild-type strain, while Robusta extended the chronological lifespan only slightly and with statistically significant results only on day 4 (Figure 3A). We found a similar data dependency in the case of the *sod1Δ* (Figure 3B) and *sod2Δ* (Figure 3C) mutants. Interestingly, in the case of the *rad52Δ* mutant (Figure 3D), the survival curves for cells treated by Arabica and Robusta were similar, while at day 21, survival was significantly higher compared to the control (*p* < 0.001). It must be noted that we did not observe a significant effect of caffeine on aging, which suggests that polyphenols had the primary role in protecting cells from damage.

Additionally, we observed the adaptative regrowth phenomenon. Regrowth is an adaptive response that enables cell subpopulation to escape from quiescence and re-enter the cell cycle [26,27]. The ability of cells to re-enter the cell cycle during chronological aging was observed for all strains treated by coffee (marked with an arrow on Figure 3).

### 2.5. Coffee Protects Cells from Oxidative Stress and DNA Damage

One of the markers by which we could demonstrate at the cellular level that coffee is a free radical scavenger is the Yap1-GFP fusion protein.

**Figure 4:**
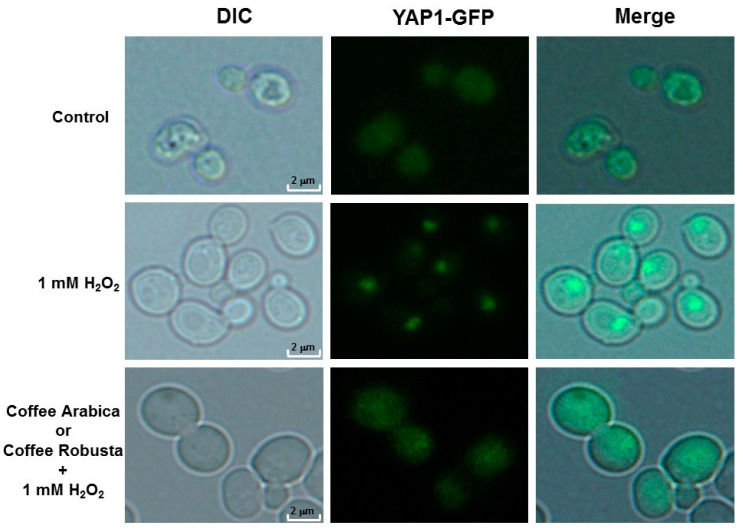
Coffee infusions protect by Yap1-GFP nuclear localization during oxidative stress inducing by hydrogen peroxide. Control—cells from the Yeast Extract–Peptone–Dextrose (YPD) culture and 1 mM H_2_O_2_—cells incubated for 1 h with hydrogen peroxide in the YPD medium. Yap1-GFP fusion protein can be seen to concentrate in the nuclei and coffee + H_2_O_2_—cells incubated for 3 h with coffee, centrifuged and hanging in fresh medium, and treated with hydrogen peroxide for 1 h. Representative results from three independent experiments are shown. Fluorescence pictures were taken with an Olympus BX-51 microscope equipped with a DP-72 digital camera and cellSens Dimension software (1000× magnification).

Yap1p is a transcription factor accumulated in the cell nucleus during oxidative stress. As shown in Figure 4, the Yap1-GFP fusion protein is located in the cell nucleus in the case of positive control after treatment with hydrogen peroxide. 

**Figure 5:**
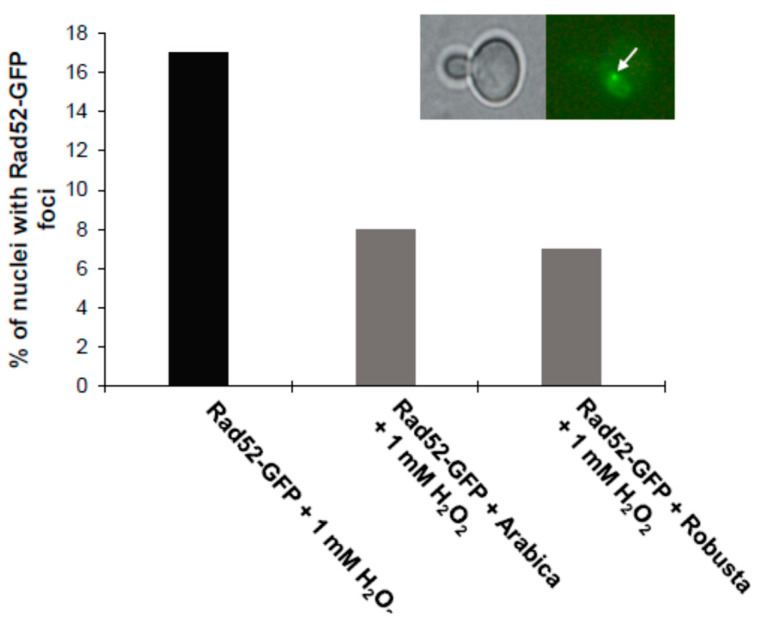
Induction of nuclear Rad52-GFP foci by 1 mM hydrogen peroxide. A sample photo of the Rad52-GFP is presented at the top (white arrow in the figure’s caption indicate foci). The results represent values for cells tested in two independent experiments (a total of 200 cells). Fluorescence pictures were taken with an Olympus BX-51 microscope equipped with a DP-72 digital camera and cellSens Dimension software (1000× magnification).

On the other hand, when cells were grown on a coffee infusion medium, they were protected against free radicals, with the transcription factor residing in cytosol after hydrogen peroxide treatment. This result shows clearly that coffee has a beneficial effect on cells through its free radical scavenging activity. We also showed that coffee protects cells by preventing DNA double-strand breaks. For this purpose, we used another marker, the Rad52-GFP protein. As shown in Figure 5, coffee reduces the number of DNA double-strand breaks as observed from the formation of Rad52-GFP foci.

### 2.6. Impact of Coffee on Sensitivity to Stress Factors

In the next step, general metabolic analyses were conducted to show the reaction of mutants and wild-type strains treated or untreated with coffee to various environmental changes, inducing intracellular acidification, cell disorder or DNA damage. The phenotype screening analysis of the yeast mutants was performed toward various metabolic conditions (Table 3). The obtained information suggests that coffee infusion has some role in adaptation of the yeast cell to the changing environmental conditions. We showed that coffee could have a protective effect against certain stress factors such as factors causing cell wall disorder (Congo red, calcofluor white) or the DNA alkylating agent methyl methanesulfonate (MMS), especially in *sod1Δ*. On the other hand, coffee, especially Robusta, may be a factor increasing sensitivity to osmotic stress (NaCl) and acetic acid, which at the toxic level inhibits yeast cell growth by impeding cell metabolic functions through intracellular acidification.

### 2.7. Impact of Coffee on the Metabolic Activity of Yeast

**Figure 6:**
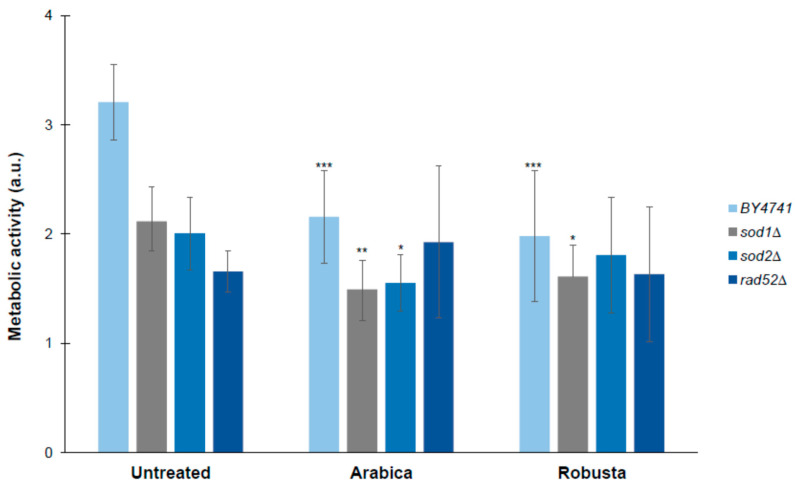
Metabolic activity of the cells (red/green ratio) was estimated with FUN-1 stain. Data are expressed as ratio of red (λ = 575 nm) to green (λ = 535 nm) fluorescence and presented as mean ± SD from three independent experiments. Bars indicate SD. Statistical significance was assessed using ANOVA and the Dunnett’s post hoc test (* *p* < 0.05; ** *p* < 0.01, *** *p* < 0.001) compared to the control (untreated).

We also showed that yeast cells treated with both Arabica and Robusta coffee infusions had a significantly reduced level of metabolism in the wild-type and *sod1Δ* strains. In turn, in the case of *sod2Δ*, metabolic activity was significantly reduced when cells were treated with the Arabica infusion. Interestingly, none of the infusions affected the metabolic activity of *rad52Δ* (Figure 6).

## 3. Discussion

In 2016, the International Agency for Research on Cancer began looking into the available body of research investigating the links between consumption of coffee and neoplastic diseases. Initially, no visible relationship was noted between such consumption and the risk of cancer developing in any of the human organs. However, it was noted in some cases that coffee consumption is positively correlated with lower occurrence of certain types of neoplasms [28]. This may be attributed to diterpenes and polyphenols, which inhibit genotoxic impact of numerous carcinogens. Moreover, there were data showing that consumption of coffee has a positive impact in the case of cardiovascular diseases. It was demonstrated that drinking approximately 3 cups of coffee per day reduces the risk of such diseases by up to 21% [29]. The age-related loss of motor skills and neuron capacity and loss of cognitive abilities are a result of the aging organism’s inability to protect itself against inflammation and oxidative stress [29]. As a complex multifactorial process, aging reduces the organism’s ability to respond to environmental stress. This leads to accumulation of intracellular damage and impairment of the tissue and organ functions, which ultimately leads to the organism’s death. One of the theories that try to explain the phenomenon of aging is the so called free radical theory of aging (FRTA). This theory attributes the destructive impact on the organism to oxidants, in particular reactive oxygen species (ROS), which cause oxidation of cellular macromolecules and therefore impair their function. The damage caused by free radicals can be counteracted by compounds capable of sweeping free radicals or transforming them into their inactive forms [23]. In older cells, in which oxidative stress gradually intensifies, increased activity of NADH dehydrogenase and cytochrome oxidase has been observed. It has also been demonstrated that reactive oxygen species accelerate telomere shortening and cell cycle inhibition [30].

Coffee beans are an important source of dietary antioxidants and coffee is one of the most antioxidant-rich beverages consumed regularly in high amounts [31,32]. The highest antioxidant capacity was noted in the coffee leaves and beans in comparison to the plant’s stem and roots. Moreover, roasted beans had significantly higher antioxidant power than the green ones [33]. The results obtained by Górecki and Hallmann [34] demonstrated that coffee’s antioxidant properties are influenced by coffee origin (organic or conventional), beans roasting level and duration of infusion. Our results confirmed previous reports that Robusta has higher antioxidant properties [35,36]. Depending on the beans roasting level, the antioxidant activity determined through the ABTS* assay ranged from 452 to 547 μmol Trolox/g for Robusta and from 108 to 327 μmol Trolox/g for Arabica, which is similar to our results. Furthermore, with a higher coffee roasting degree the antioxidant activity measured with the FRAP method decreased for both coffee types [35]. The slight difference observed for the ABTS and FRAP results was probably caused by the mechanism used in antioxidant power estimation, which is restricted to measuring compounds that promote electron transfer [36]. Additionally, we demonstrated that caffeine has no antioxidant effect, simultaneously samples with high antioxidant activity contained more polyphenols, especially chlorogenic acid isomers. Determined polyphenolic profiles of coffee infusions were typical for coffee beverages reported by other authors [37,38].

In this study the *S. cerevisiae* budding yeast was used as a model to test whether a specialty coffee infusion has an antiaging effect on cells with free radical detoxification impairment (*SOD1* and *SOD2* mutants) and DNA thread repair impairment (*RAD52* mutant). Analysis of the cell survival rate was conducted with the use of the standard chronological aging method. This method allows for successful analysis of cell aging in the post-mitotic phase; it should be noted that this is an acceptable model of studying aging of non-dividing cells in higher eukaryotes, including humans. Results of analyses performed during the study clearly suggest that coffee infusion, regardless of the coffee type, statistically significantly extends cell life. This is due to the high content of substances with antioxidant properties. Interestingly, although the impact of coffee as such was documented in other experimental setups, the antioxidant effect of caffeine as the main ingredient associated with coffee has not been confirmed yet.

Some recent data show that caffeine extended yeast lifespan via inhibition of the evolutionary conserved kinase TORC1-Sch9, which suggests that caffeine may extend lifespan in other eukaryotes, including humans [39]. Inhibition of Tor1p through deletion or in pharmacological manner, e.g., by rapamycin, has been known to extend the lifespan of yeast and many other organisms [40,41,42]. However, we showed that cellular metabolic activity was reduced when compared to cells untreated with coffee. Cellular metabolism is regulated by the mTOR kinase, a key component of the molecular nutrient sensor pathway that plays a central role in cellular survival and aging. Therefore, it is probable that all bioactive components of coffee act in a synergistic manner in both antioxidative and metabolic regulation. A previous study involving rats showed that the neuroprotective benefits of coffee are not due to caffeine alone, but rather to other bioactive compounds in coffee [43]. Furthermore, it was concluded that the neuroprotective benefits of coffee are not due to caffeine alone but should probably be attributed to polyphenols and other bioactive compounds of coffee. In particular, these bioactive molecules may complement or synergize with caffeine to produce their beneficial actions [44]. We did not show a positive effect of caffeine on aging in this experimental setup (data not shown), which is consistent with the data on the caffeine’s lack of antioxidant capacity.

In order to prove the antioxidant action of coffee, a test was conducted in which oxidative stress was induced with hydrogen peroxide in the strain with the Yap1-GFP fusion marker protein. Yap1 protein is a member of the AP-1 transcription factors family activating antioxidant gene transcription in response to oxidative stress [45]. Yap1 is involved in one of the three mechanisms regulating the transcription response to oxidative stress. With the lack of oxidative stress, Yap1p is exported from the nucleus by exportin to reside in cytosol [46]. During oxidative stress induction, formation of the intramolecular disulfide bond in Yap1 is catalyzed in the presence of hydrogen peroxide. Due to this conformational change Yap1 may be accumulated in the cell nucleus [47]. During our study we conducted the Yap1-GFP transcription factor activation test. The results of our analysis show clearly that coffee infusion provides cells with effective protection against oxidative stress. These findings concord with previous scientific reports suggesting that polyphenols in coffee determine the beverage’s strong antioxidant action [48]. Surprisingly, some of the data suggest that caffeine may have both antioxidant and prooxidant effects [49]. Latest reports show that caffeine has an impact on extending lifespan of *Caenorhabditis elegans* in a dose-dependent way [50,51,52]. What is interesting, plant extracts containing mainly caffeine have been shown to play a positive part in extending the lifespan of *C. elegans* [53].

In addition, we conducted a test confirming that coffee protects yeast cells against DNA strand breaks. The *RAD52* gene product is involved in the repair of DNA double-strand breaks (DSBs). After inducing DNA double-strand breaks by, e.g., gamma radiation, Rad52-GFP move from the nuclear diffuse form to separate clusters known as foci [54]. The *rad52∆* mutant used for the analysis is defective in terms of repairing DNA damage caused by physical or chemical factors, as shown in the MMS toxicity tests. The Rad52-GFP fusion protein, on the other hand, is fully biologically functional for the purpose of DNA reparation and recombination. The analysis shows that when oxidative stress is induced with the use of hydrogen peroxide, fewer Rad52-GFP fusion protein foci are formed in comparison to the control. This may lead to a conclusion that coffee infusion provides protection to the cell on many levels, including helping the cell maintain genome stability, which is probably achieved by reducing the oxygen free radical amount in the cell.

Aging is an irreversible process that affects cell physiology and metabolism. Earlier analyses have shown that the process may be slowed down in yeast by reducing the cell’s metabolic rate [55]. For this purpose, analysis of cell metabolic activity was performed with the use of the FUN-1 fluorescent dye. It was observed that cells grown on a medium with coffee infusion added have a significantly lower metabolic activity. We therefore suggest that coffee may be beneficial to yeast cells not only due to detoxification of free oxygen radicals but also due to reduction of the metabolism rate. On the other hand, it has been suspected that the main determinant of aging delay is reduction of the metabolic rate, which subsequently leads to lowering of the cell’s ROS level. Moreover, reducing the overall metabolic activity might contribute to extending of the chronological lifespan due to slower production of secondary metabolites, e.g., acetic acid, which potentially may have an impact on aging [56,57].

Coffee has many properties that are advantageous to human health as confirmed in clinical research as well as cell-based and animal-based studies. It has been observed that coffee has a beneficial effect on reduction of inflammatory markers, obesity, risk of developing type 2 diabetes, hypertension and atherosclerosis [58,59,60]. Moreover, the Arabica coffee shows chemoprotective properties by promoting cytotoxicity followed by cell cycle arrest in phase S and apoptosis induction in cancer cells [61]. Results of many studies of acute and long-term human interventions suggested that coffee consumption had an influence on the modulation of oxidative stress. It was observed that intake of coffee could increase the glutathione level, antioxidant capacity of plasma and antioxidant enzyme activity and improve protection against DNA and lipid damage [62,63,64,65,66].

## 4. Materials and Methods

### 4.1. Preparation of Coffee Infusions

For the purpose of the study, two types of coffee Arabica and Robusta of different origin (Arabica comes from Ethiopia–Yirgacheffe; light roast), while Robusta comes from Panama—Penonome; medium roast) and originating from different botanical species were used; they were also obtained from different coffee roasters. The coffee types used in the study were of specialty grade quality. Appropriate amount of each coffee was weighed for each infusion preparation method. The coffee was ground in the Comandante C40 MK3 Nitro Blade grinder (Germany). Infusion was prepared immediately after grinding with the use of distilled water.

### 4.2. Preparing Infusion with a Dripper

Prefiltered water was heated to 90 ˚C in the Brewista Artisan Gooseneck electric kettle. In the meantime, 12 g of coffee beans were weighed using the Acaia Pearl scales with up to 0.1 g accuracy and ground in the Comandante C40 MK3 Nitro Blade grinder at 20 clicks. The Hario (Japan) V60-02 dripper (ceramic cone with the porous bottom) with Hario V60-02 VCF-02 100 W paper filter was placed on the Hario Coffee Server V60-02 700 mL (glass jug for collecting the infusion) and slushed with hot water in order to warm up the dripper and server, and eliminate the paper aftertaste from the infusion. The water was removed from the server; the ground coffee was put into the filter, poured over with the first portion of water (24 g) and left for 30 s—a so called preinfusion during which coffee absorbed water releasing carbon dioxide. After 30 s 176 g of water was added; the staff waited until all the water went through the ground coffee beans. The total brewing time was 2 min. All preparing infusion was repeated six times.

### 4.3. Determination of Caffeine and Polyphenols Concentration in Coffee Infusions Using HPLC

Chromatographic analyses were carried out in Laboratory of Plant Biotechnology “Aeropolis” using the Gilson chromatographic set (Gilson, Middleton, Wisconsin, USA), equipped with binary pump (Gilson 322), DAD (Diode-Array Detection) detector (Gilson 172), column thermostat (Knauer, Berlin, Germany) and autosampler with fraction collector (GX-271 Liquid Handler).

The HPLC method for caffeine determination carried out according to Fajara and Susanti (2017) [67] with minor modifications. The separation was performed using Knauer Nucleosil II C-18 100-5 column (250 mm × 4.6 mm) with precolumn (Gilson). The elution was performed isocratically with water-methanol (95–5, *v/v*) mobile phase, flow rate: 1 mL∙min^-1^, and time of analysis: 7 min. The injection volume of samples (previously filtered through 0.22 μm nylon filters) was 10 µL. Analyte was detected at wavelength 272 nm. For quantitative analysis of caffeine the standard curve was prepared using standard solution within the concentration range 0.0625–1 mg (y = 18940x + 944.19, R^2^ = 0.9881). The LOQ (Limit of Quantitation) and LOD (Limit of Detection) were found to be 2 and 0.5 μg/mL respectively.

The separation of polyphenols was carried out using Poroshell 120 EC-C18 column (150 mm × 4.6 mm) with the precolumn (Agilent). The mobile phase was a mixture of 0.1% HCOOH (A) and acetonitrile (B). A gradient elution program was used: linear gradient to 10% B in the first 1.55 min, linear gradient from 10 to 100% B in the next 1.55–20 min and 100% B in the next 20–25 min. The flow rate of the mobile phase was 1 mL/min. Detection of individual compounds was performed at 230, 254, 280, 320 and 360 nm. The injection volume of the sample (previously filtered through 0.22 μm nylon filters) was 20 μL.

The quantitative analysis of phenolic acids in the extracts was carried out on the basis of the prepared standard curve for chlorogenic acid in the concentration range 10−500 μg/mL (y = 41.761x − 1374, R^2^ = 0.9987). The LOQ and LOD were found to be 1 and 0.1 μg/mL respectively.

### 4.4. Determination by the ABTS Radical Method

The antioxidant activity was determined using the free radical ABTS (2,2′-azino-bis (3-ethylbenzothiazoline-6-sulphonic acid)) [68]. The ABTS* was formed by the reaction of ABTS solution 7 mM with 2.45 mM of potassium persulfate, and incubated for 24 h at room temperature and protected from light. Briefly, appropriate amounts of coffee infusion diluted at a concentration of 1:20 were added to a solution of ABTS*, diluted such that 200 µL of the solution had absorbance of 1.0 ± 0.04 in a microplate well. The reading of absorbance was done in the Tecan Infinite 200 PRO multimode reader (Tecan Group Ltd., Männedorf, Switzerland) at 734 nm after six minutes of reaction in room temperature. Ethanol solutions with known concentrations of Trolox were used for calibration. Results were expressed in Trolox equivalents (μmol Trolox/g of sample). All measurements were repeated three times.

### 4.5. Determination by the FRAP Assay

Antioxidant capacity was determined using the FRAP assay (ferric reducing antioxidant potential) with 0.3 M acetate buffer (pH = 3.6), 0.01 M TPTZ (2,4,6-tripyridyl-s-triazine) in 0.04 M HCl and 0.02 M FeCl_3_*6H_2_O mixed in 10:1:1 and coffee infusion [69]. Absorbance was done in the Tecan Infinite 200 PRO multimode reader (Tecan Group Ltd., Männedorf, Switzerland) at 593 nm after 20 min incubation in room temperature. Ethanol solutions with known concentrations of Trolox were used for calibration. Results were expressed in Trolox equivalents (μmol Trolox/g of sample). All measurements were repeated three times.

### 4.6. Determination of DPPH Free Radical Scavenging Activity

DPPH (2,2-diphenyl-1-picrylhydrazyl) radical inhibition was measured according to the assay described by Blois (1958) [70] with minor modifications. The aliquot of 20 μL of sample solution (diluted 50×) was mixed with 180 μL of DPPH radical methanolic solution (0.1 mM) and kept in the dark for 30 min. After incubation, the absorbance of the tested and control samples was measured at 517 nm against methanol using EPOCH 2 microplate reader (Biotek, Winooski, VT, USA). The reduction of DPPH radicals was calculated using the following equation:

AA% = [(A_0_ ×A_s_)/A_0_] × 100 where A_0_ is the absorbance of control and A_s_ is the absorbance of the tested samples.

### 4.7. Total Phenolic Content

The total phenolics content was determined using a Folin–Ciocalteu reagent, according to Singleton and Rossi (1965) [71] with minor modifications. To 20 μL of sample solution (diluted 50×), 100 μL of 10% Folin–Ciocalteu reagent followed by 80 μL of 7.5% (*w/v*) of Na_2_CO_3_ solution were added. Samples were kept in the dark for 120 min and the absorbance was then measured against blank at 760 nm using EPOCH 2 microplate reader (Biotek, Winooski, VT, USA). The phenolic content expressed as milligram of gallic acid (GAE) equivalents per milliliter (mg GAE/mL) was calculated based on a calibration curve prepared for gallic acid in the range of 0−250 μg/mL (y = 0.0336x, r^2^ = 0.9914).

### 4.8. Strains and Growth Conditions

The following yeast strains were used: wild type strain BY4741 (MATa; his3; leu2; met15; ura3), *sod1Δ* (MATa; his3; leu2; met15; ura3; YJR104C::kanMX4), *sod2Δ* (MATa; his3; leu2; met15; ura3; YHR008C::kanMX4C) and *rad52Δ* (MATa; his3; leu2; met15; ura3; YML032C::kanMX4; Euroscarf; Germany). Yeast cells were grown in a standard liquid Yeast Extract–Peptone–Dextrose (YPD) medium (1% Difco yeast extract, 1% yeast bacto-peptone and 2% glucose) on a rotary shaker at 150 rpm or on solid YPD medium containing 2% agar. The experiments were carried out at 28 °C.

### 4.9. Kinetics of the Growth Assay

The growth assays were carried out on liquid medium described elsewhere. The yeast cells suspension was incubated for 12 h in a shaking incubator at 28 °C (Heidolph Incubator 1000 at 1200 rpm (Germany)). The growth was monitored in the Anthos 2010 type 17550 microplate reader (Austria) at 600 nm by measurements at 2 h intervals for 12 h. For each experiment, an infusion volume equal to 0.6 mg/mL of the determined caffeine concentration was taken.

### 4.10. Chronological Life Span (CLS) Assays

Chronological life span of cells incubated in minimal medium (SDC) was measured as described previously [25]. Briefly, yeast were grown in SDC containing 2% (*w/v*) glucose, supplemented with histidine, leucine, methionine, uracil and appropriate concentration of caffeine and coffee. For each experiment, an infusion volume equal to 0.6 mg/mL of the determined caffeine concentration was taken. Chronological life span was monitored in expired SDC medium by measuring viability in 2, 5, 8, 12, 19 and 26 days. For the quantitative measurement of survival, staining with propidium iodide was used. The data represent the mean values from three independent experiments.

### 4.11. Cell Viability

For determining death cells staining with propidium iodide was used. Cells were suspended in PBS and stained with 5 mg/mL propidium iodide (Sigma-Aldrich) for 15 min in the dark at room temperature. Fluorescence pictures were taken with Olympus BX-51 microscope equipped with a DP-72 digital camera and cellSens Dimension software. Dead cells were red fluorescent (λex = 480 nm; λem = 520 nm). The data represent the mean values from three independent experiments.

### 4.12. Phenotypic Analysis—A Spot Test for Sensitivity to Congo Red, Calcofluor White, Methyl Methanesulfonate (MMS), Sodium Chloride (NaCl) and Acetic Acid

Yeast cultures were grown to exponential phase (OD_600nm_ between 0.8 and 1) and serially diluted to different cellular concentrations as indicated. Five microliters of each cell suspension was spotted onto agar plates containing coffee infusions or not (control) and various concentrations of Congo red (Sigma-Aldrich), Calcofluor white (Sigma-Aldrich), methyl methanesulfonate (Sigma-Aldrich), sodium chloride (NaCl; Sigma-Aldrich) and acetic acid (Sigma-Aldrich). Growth was registered 48 h after incubation at 30 °C. For each experiment, an infusion volume equal to 0.6 mg/mL of the determined caffeine concentration was taken. All phenotypes described in this work were confirmed by three independent tests.

### 4.13. Measurement of Cell Metabolic Activity

Metabolic activity of yeast cells was assessed with FUN-1 according to the manufacturer protocol (Molecular Probes) with modification described by Kwolek-Mirek and Zadrag-Tecza [72]. The fluorescence of the cell suspension was measured after 15 min incubation in the dark and at 28 °C using the TECAN Infinite 200 microplate reader (Grodig, Austria) at λ_ex_ = 480 nm and λ_em_ = 500–650 nm. The metabolic activity of cells was expressed as a change in ratio of red (λ = 575 nm) to green fluorescence (λ = 535 nm). Cell observations were also carried out using a fluorescence microscope Olympus BX-51 (Tokyo, Japan) equipped with a digital camera DP-72 and software cell^D.

### 4.14. Cellular Localization of the Yap1-GFP and Rad52-GFP Proteins

Arabica and Robusta coffee infusions at a concentration of 0.3 mg/mL caffeine were added to a yeast cell suspension at a density of 1 × 10^6^ cells/mL. Incubated at 28 °C with shaking, then cells were washed twice in PBS and added hydrogen peroxide at a final concentration of 1 mM and incubated an hour at 28 °C with shaking. Observations of Yap1-GFP localization and Rad52-GFP foci were made using an Olympus BX-51 (Tokyo, Japan) microscope (λex = 488 nm, λem = 510 nm).

### 4.15. Statistical Analysis

The results represent the mean ± SD values for all cells tested in two independent experiments. The differences between the wild-type and the isogenic mutant strains were estimated using one-way ANOVA and Dunnett’s post-hoc tests or Tukey’s honestly significant difference test. The values were considered significant when * *p* < 0.05; ** *p* < 0.01, *** *p* < 0.001. The statistical analysis was performed using the Statistica 10 software.

## Figures and Tables

**Table 1:** Average caffeine and polyphenol content in Arabica and Robusta infusions.

Compound	Average Retention Time (min)	Quantity	
		Arabica	Robusta
**Caffeine (mg/mL)**			
**caffeine**	3.5	1.63	2.55
**Polyphenols (μg/mL)**			
**neochlorogenic acid**	2.56	79.07	167.21
**cryptochlorogenic acid**	3.87	224.34	277.41
**chlorogenic acid**	4.22	225.21	150.49
**ferulic acid**	6.58	35.13	110.18
**dicaffeoylquinic or feruloylquinic acids isomers**	6.82	5.06	7.28
	6.99	12.85	22.72
	7.16	11.06	16.93
	8.07	3.66	11.68
	8.33	1.61	7.16
	8.60	7.19	25.98

**Table 2:** Total phenolic content and antioxidant capacity of Arabica and Robusta coffee infusions.

Method	Robusta Coffee	Arabica Coffee	Caffeine 1 mg/mL	Caffeine 10 mg/mL
**TPC (mg GAE/mL)**	3.11 ± 0.22 ^b^	2.08 ± 0.07 ^a^	0	0
**ABTS (μmol Trolox/g of sample)**	413.49 ± 11.26 ^b^	258.41 ± 5.56 ^a^	0	0
**FRAP (μmol Trolox/g of sample)**	392.87 ± 17.75 ^b^	262.6 ± 6.73 ^a^	0	0
**DPPH (%)**	46.09 ± 2.93 ^b^	27.97 ± 3.06 ^a^	1.73 ± 0.28 ^c^	2.40 ± 0.27 ^d^

^a, b, c, d^ Means marked with different superscript letter within the column are significantly different (Tukey’s honest significant difference test, *p* < 0.05).

**Table 3:** Sensitivity of the BY4741 strain and isogenic mutant strains *sod1Δ*, *sod2Δ* and *rad52Δ* treated with Arabica or Robusta coffee infusions on cells stressed factors.

*Strain*	Growth Conditions					
	Congo Red 15 μg/mL	Congo Red 15 µg/mL + Arabica	Congo Red 15 µg/mL + Robusta	Calcofluor White 20 µg/mL	Calcofluor White 20 µg/mL + Arabica	Calcofluor White 20 µg/mL + Robusta
*BY4741*	++++	++++	+++	++++	++++	++++
*sod1Δ*	++	+++	++++	++	++++	++++
*sod2Δ*	++++	++++	+++	++++	++++	++++
*rad52Δ*	+++	++++	++++	+++	+++	++++
***Strain***	**NaCl 0.5 M**	**NaCl 0.5 M + Arabica**	**NaCl 0.5 M + Robusta**	**CH_3_COOH 40 mM**	**CH_3_COOH 40 mM + Arabica**	**CH_3_COOH 40 mM + Robusta**
*BY4741*	++++	+++	++	+++	+++	++
*sod1Δ*	++++	+++	+	++	++	+
*sod2Δ*	++++	+++	+++	+++	++++	++
*rad52Δ*	++++	+++	++	+++	++++	+++
***Strain***	**MMS 0.03%**	**MMS 0.03% + Arabica**	**MMS 0.03% + Robusta**	**Control**		
*BY4741*	+++	++++	++++	++++		
*sod1Δ*	++	++++	+++	++++		
*sod2Δ*	++++	++++	++++	++++		
*rad52Δ*	-	-	-	++++		

Cells were cultured overnight in liquid YPD medium, counted and diluted serially to obtain suspensions at the densities 10^7^, 10^6^, 10^5^ and 10^4^ cells/mL. Five microliters of each suspension was spotted at YPD solid medium containing Arabica or Robusta coffee infusions and analyzed toxic factors. Plates were cultivated 2 days in 28 °C. Each ‘‘+’’ means the growth of one spot containing respectively: 50,000, 5000, 500 and 50 cells, "-" means no growth.

## References

[1] Gunter M.J., Murphy N., Cross A.J., Dossus L., Dartois L., Fagherazzi G., Kaaks R., Kuhn T., Boeing H., Aleksandrova K. (2017). Coffee Drinking and Mortality in 10 European Countries A Multinational Cohort Study. Ann. Intern. Med..

[2] Privat I., Foucrier S., Prins A., Epalle T., Eychenne M., Kandalaft L., Caillet V., Lin C.W., Tanksley S., Foyer C. (2008). Differential regulation of grain sucrose accumulation and metabolism in *Coffea arabica* (Arabica) and *Coffea canephora* (Robusta) revealed through gene expression and enzyme activity analysis. New Phytol..

[3] Perrois C., Strickler S.R., Mathieu G., Lepelley M., Bedon L., Michaux S., Husson J., Mueller L., Privat I. (2015). Differential regulation of caffeine metabolism in *Coffea arabica* (Arabica) and *Coffea canephora* (Robusta). Planta.

[4] Navarini L., Gilli R., Gombac V., Abatangelo A., Bosco M., Toffanin R. (1999). Polysaccharides from hot water extracts of roasted *Coffea arabica* beans: Isolation and characterization. Carbohydr. Polym..

[5] Kang D.E., Lee H.U., Davaatseren M., Chung M.S. (2020). Comparison of acrylamide and furan concentrations, antioxidant activities, and volatile profiles in cold or hot brew coffees. Food Sci. Biotechnol..

[6] Spencer M., Sage E., Velez M., Guinard J.X. (2016). Using Single Free Sorting and Multivariate Exploratory Methods to Design a New Coffee Taster’s Flavor Wheel. J. Food Sci..

[7] Heckman M.A., Weil J., de Mejia E.G. (2010). Caffeine (1, 3, 7-trimethylxanthine) in Foods: A Comprehensive Review on Consumption, Functionality, Safety, and Regulatory Matters. J. Food Sci..

[8] Frischknecht P.M., Ulmerdufek J., Baumann T.W. (1986). Purine Alkaloid Formation in Buds and Developing Leaflets of *Coffea-arabica*—Expression of an Optimal Defense Strategy. Phytochemistry.

[9] Ricci E., Vigano P., Cipriani S., Somigliana E., Chiaffarino F., Bulfoni A., Parazzini F. (2017). Coffee and caffeine intake and male infertility: A systematic review. Nutr. J..

[10] Nehlig A. (2018). Interindividual Differences in Caffeine Metabolism and Factors Driving Caffeine Consumption. Pharmacol. Rev..

[11] Yang A., Palmer A., de Wit H. (2010). Genetics of caffeine consumption and responses to caffeine. Psychopharmacology.

[12] Kot M., Daniel W.A. (2008). Caffeine as a marker substrate for testing cytochrome P450 activity in human and rat. Pharmacol. Rep..

[13] Cappelletti S., Daria P., Sani G., Aromatario M. (2015). Caffeine: Cognitive and Physical Performance Enhancer or Psychoactive Drug?. Curr. Neuropharmacol..

[14] DePaula J., Farah A. (2019). Caffeine Consumption through Coffee: Content in the Beverage, Metabolism, Health Benefits and Risks. Beverages.

[15] Agostoni C., Canani R.B., Fairweather-Tait S., Heinonen M., Korhonen H., La Vieille S., Marchelli R., Martin A., Naska A., Neuhauser-Berthold M. (2015). Scientific Opinion on the safety of caffeine. EFSA J..

[16] Ferruzzi M.G. (2010). The influence of beverage composition on delivery of phenolic compounds from coffee and tea. Physiol. Behav..

[17] Takahashi K., Ishigami A. (2017). Anti-aging effects of coffee. Aging Us.

[18] Buldak R.J., Hejmo T., Osowski M., Buldak L., Kukla M., Polaniak R., Birkner E. (2018). The Impact of Coffee and Its Selected Bioactive Compounds on the Development and Progression of Colorectal Cancer In Vivo and In Vitro. Molecules.

[19] Welsh E.J., Bara A., Barley E., Cates C.J. (2010). Caffeine for asthma. Cochrane Database Syst. Rev..

[20] Roehrs T., Roth T. (2008). Caffeine: Sleep and daytime sleepiness. Sleep Med. Rev..

[21] Yadegari M., Khazaei M., Anvari M., Eskandari M. (2016). Prenatal Caffeine Exposure Impairs Pregnancy in Rats. Int. J. Fertil. Steril..

[22] Sengpiel V., Elind E., Bacelis J., Nilsson S., Grove J., Myhre R., Haugen M., Meltzer H.M., Alexander J., Jacobsson B. (2013). Maternal caffeine intake during pregnancy is associated with birth weight but not with gestational length: Results from a large prospective observational cohort study. BMC Med..

[23] Harman D. (1956). Aging—A Theory Based on Free-Radical and Radiation-Chemistry. J. Gerontol..

[24] Kaeberlein M. (2010). Lessons on longevity from budding yeast. Nature.

[25] Fabrizio P., Longo V.D. (2003). The chronological life span of Saccharomyces cerevisiae. Aging Cell.

[26] Fabrizio P., Pletcher S.D., Minois N., Vaupel J.W., Longo V.D. (2004). Chronological aging-independent replicative life span regulation by Msn2/Msn4 and Sod2 in Saccharomyces cerevisiae. Febs Lett..

[27] Longo V.D., Shadel G.S., Kaeberlein M., Kennedy B. (2012). Replicative and Chronological Aging in Saccharomyces cerevisiae. Cell Metab..

[28] Loomis D., Guyton K.Z., Grosse Y., Lauby-Secretan B., El Ghissassi F., Bouvard V., Benbrahim-Tallaa L., Guha N., Mattock H., Straif K. (2016). Carcinogenicity of drinking coffee, mate, and very hot beverages. Lancet Oncol..

[29] Crippa A., Discacciati A., Larsson S.C., Wolk A., Orsini N. (2014). Coffee Consumption and Mortality from All Causes, Cardiovascular Disease, and Cancer: A Dose-Response Meta-Analysis. Am. J. Epidemiol..

[30] Von Zglinicki T. (2000). Role of oxidative stress in telomere length regulation and replicative senescence. Mol. Cell. Gerontol..

[31] Yashin A., Yashin Y., Wang J.Y., Nemzer B. (2013). Antioxidant and Antiradical Activity of Coffee. Antioxidants.

[32] Janda K., Jakubczyk K., Baranowska-Bosiacka I., Kapczuk P., Kochman J., Rebacz-Maron E., Gutowska I. (2020). Mineral Composition and Antioxidant Potential of Coffee Beverages Depending on the Brewing Method. Foods.

[33] Acidri R., Sawai Y., Sugimoto Y., Handa T., Sasagawa D., Masunaga T., Yamamoto S., Nishihara E. (2020). Phytochemical Profile and Antioxidant Capacity of Coffee Plant Organs Compared to Green and Roasted Coffee Beans. Antioxidants.

[34] Gorecki M., Hallmann E. (2020). The Antioxidant Content of Coffee and Its In Vitro Activity as an Effect of Its Production Method and Roasting and Brewing Time. Antioxidants.

[35] Pokorna J., Venskutonis P.R., Kraujalyte V., Kraujalis P., Dvorak P., Tremlova B., Kopriva V., Ostadalova M. (2015). Comparison of Different Methods of Antioxidant Activity Evaluation of Green and Roast *C. arabica* and *C. robusta* Coffee Beans. Acta Aliment..

[36] Vignoli J.A., Viegas M.C., Bassoli D.G., Benassi M.D. (2014). Roasting process affects differently the bioactive compounds and the antioxidant activity of arabica and robusta coffees. Food Res. Int..

[37] Stalmach A., Mullen W., Nagai C., Crozier A. (2006). On-line HPLC analysis of the antioxidant activity of phenolic compounds in brewed, paper-filtered coffee. Braz. J. Plant Physiol..

[38] Belguidoum K., Amira-Guebailia H., Boulmokh Y., Houache O. (2014). HPLC coupled to UV-vis detection for quantitative determination of phenolic compounds and caffeine in different brands of coffee in the Algerian market. J. Taiwan Inst. Chem. Eng..

[39] Wanke V., Cameroni E., Uotila A., Piccolis M., Urban J., Loewith R., De Virgilio C. (2008). Caffeine extends yeast lifespan by targeting TORC1. Mol. Microbiol..

[40] Molon M., Zadrag-Tecza R., Bilinski T. (2015). The longevity in the yeast Saccharomyces cerevisiae: A comparison of two approaches for assessment the lifespan. Biochem. Biophys. Res. Commun..

[41] Hwangbo D.S., Lee H.Y., Abozaid L.S., Min K.J. (2020). Mechanisms of Lifespan Regulation by Calorie Restriction and Intermittent Fasting in Model Organisms. Nutrients.

[42] Selvarani R., Mohammed S., Richardson A. (2020). Effect of rapamycin on aging and age-related diseases-past and future. Geroscience.

[43] Shukitt-Hale B., Miller M.G., Chu Y.F., Lyle B.J., Joseph J.A. (2013). Coffee, but not caffeine, has positive effects on cognition and psychomotor behavior in aging. Age.

[44] Cao C.H., Wang L., Lin X.Y., Mamcarz M., Zhang C., Bai G., Nong J., Sussman S., Arendash G. (2011). Caffeine Synergizes with Another Coffee Component to Increase Plasma GCSF: Linkage to Cognitive Benefits in Alzheimer’s Mice. J. Alzheimers Dis..

[45] Toone W.M., Jones N. (1999). AP-1 transcription factors in yeast. Curr. Opin. Genet. Dev..

[46] Yan C., Lee L.H., Davis L.I. (1998). Crm1p mediates regulated nuclear export of a yeast AP-1-life transcription factor. Embo Jl.

[47] Gulshan K., Rovinsky S.A., Coleman S.T., Moye-Rowley W.S. (2005). Oxidant-specific folding of Yap1p regulates both transcriptional activation and nuclear localization. J. Biol. Chem..

[48] Chu Y.F., Brown P.H., Lyle B.J., Chen Y.M., Black R.M., Williams C.E., Lin Y.C., Hsu C.W., Cheng I.H. (2009). Roasted Coffees High in Lipophilic Antioxidants and Chlorogenic Acid Lactones Are More Neuroprotective than Green Coffees. J. Agric. Food Chem..

[49] Azam S., Hadi N., Khan N.U., Hadi S.M. (2003). Antioxidant and prooxidant properties of caffeine, theobromine and xanthine. Med Sci. Monit. Int. Med. J. Exp. Clin. Res..

[50] Li H.M., Roxo M., Cheng X.L., Zhang S.X., Cheng H.R., Wink M. (2019). Pro-oxidant and lifespan extension effects of caffeine and related methylxanthines in Caenorhabditis elegans. Food Chem. X.

[51] Bridi J.C., Barros A.G.D., Sampaio L.R., Ferreira J.C.D., Soares F.A.A., Romano-Silva M.A. (2015). Lifespan Extension Induced by Caffeine in Caenorhabditis elegans is Partially Dependent on Adenosine Signaling. Front. Aging Neurosci..

[52] Sutphin G.L., Bishop E., Yanos M.E., Moller R.M., Kaeberlein M. (2012). Caffeine extends life span, improves healthspan, and delays age-associated pathology in Caenorhabditis elegans. Longev. Healthspan.

[53] Peixoto H., Roxo M., Krstin S., Roehrig T., Richling E., Wink M. (2016). An Anthocyanin-Rich Extract of Acai (*Euterpe precatoria* Mart.) Increases Stress Resistance and Retards Aging-Related Markers in Caenorhabditis elegans. J. Agric. Food Chem..

[54] Lisby M., Rothstein R., Mortensen U.H. (2001). Rad52 forms DMA repair and recombination centers during S phase. Proc. Natl. Acad. Sci. USA.

[55] Molon M., Szajwaj M., Tchorzewski M., Skoczowski A., Niewiadomska E., Zadrag-Tecza R. (2016). The rate of metabolism as a factor determining longevity of the Saccharomyces cerevisiae yeast. Age.

[56] Mirisola M.G., Longo V.D. (2012). Acetic acid and acidification accelerate chronological and replicative aging in yeast. Cell Cycle.

[57] Burhans W.C., Weinberger M. (2009). Acetic acid effects on aging in budding yeast. Cell Cycle.

[58] Yamagata K. (2018). Do Coffee Polyphenols Have a Preventive Action on Metabolic Syndrome Associated Endothelial Dysfunctions? An Assessment of the Current Evidence. Antioxidants.

[59] Liang N.J., Kitts D.D. (2014). Antioxidant Property of Coffee Components: Assessment of Methods that Define Mechanisms of Action. Molecules.

[60] Patay E.B., Bencsik T., Papp N. (2016). Phytochemical overview and medicinal importance of Coffea species from the past until now. Asian Pac. J. Trop. Med..

[61] Gasscht F., Dicato M., Diederich M. (2015). Coffee provides a natural multitarget pharmacopeia against the hallmarks of cancer. Genes Nutr..

[62] Martini D., Del Bo C., Tassotti M., Riso P., Del Rio D., Brighenti F., Porrini M. (2016). Coffee Consumption and Oxidative Stress: A Review of Human Intervention Studies. Molecules.

[63] Kotyczka C., Boettler U., Lang R., Stiebitz H., Bytof G., Lantz I., Hofmann T., Marko D., Somoza V. (2011). Dark roast coffee is more effective than light roast coffee in reducing body weight, and in restoring red blood cell vitamin E and glutathione concentrations in healthy volunteers. Mol. Nutr. Food Res..

[64] Steinkellner H., Hoelzl C., Uhl M., Cavin C., Haidinger G., Gsur A., Schmid R., Kundi M., Bichler J., Knasmuller S. (2005). Coffee consumption induces GSTP in plasma and protects lymphocytes against (+/-)-anti-benzo a pyrene-7,8-dihydrodiol-9,10-epoxide induced DNA-damage: Results of controlled human intervention trials. Mutat. Res Fundam. Mol. Mech. Mutagen..

[65] Yukawa G.S., Mune M., Otani H., Tone Y., Liang X.M., Iwahashi H., Sakamoto W. (2004). Effects of coffee consumption on oxidative susceptibility of low-density lipoproteins and serum lipid levels in humans. Biochem. Mosc..

[66] Cardin R., Piciocchi M., Martines D., Scribano L., Petracco M., Farinati F. (2013). Effects of coffee consumption in chronic hepatitis C: A randomized controlled trial. Dig. Liver Dis..

[67] Fajara B.E.P., Susanti H. (2017). HPLC determination of caffeine in coffee beverage. Conf. Ser. Mater. Sci. Eng..

[68] Re R., Pellegrini N., Proteggente A., Pannala A., Yang M., Rice-Evans C. (1999). Antioxidant activity applying an improved ABTS radical cation decolorization assay. Free Radic. Biol. Med..

[69] Benzie I.F.F., Strain J.J. (1996). The ferric reducing ability of plasma (FRAP) as a measure of ‘‘antioxidant power’’: The FRAP assay. Anal. Biochem..

[70] Blois M.S. (1958). Antioxidant determinations by the use of a stable free radical. Nature.

[71] Singleton V.L., Rossi J.A. (1965). Colorimetry of total phenolics with phosphomolybdic-phosphotungstic acid reagents. Am. J. Enol. Viticult..

[72] Kwolek-Mirek M., Zadrag-Tecza R. (2014). Comparison of methods used for assessing the viability and vitality of yeast cells. FEMS Yeast Res..

